# Posterior urethral valves with vesical calculus: A rare association

**DOI:** 10.4103/0971-9261.57705

**Published:** 2009

**Authors:** Himanshu Acharya, N. P. Dhende, S. B. Mane, Abu Obaidah

**Affiliations:** Department of Paediatric Surgery, SIR J J Group of Hospitals, Mumbai, India

**Keywords:** Bladder outlet obstruction, posterior urethral valves, urethral Calculi, vesical calculi

## Abstract

Posterior urethral valve and vesical calculus are individually among the most common causes of obstructive lower urinary complaints in children. There are very few reports of association between posterior urethral valves and bladder calculus. We report three such cases. This association of the vesical calculi with posterior urethral valves may lead to a delay in the diagnosis of the posterior urethral valves. The diagnosis of posterior urethral valves should be suspected in all children with vesical or urethral calculi who have persistence of symptoms even after removal of the calculi.

## INTRODUCTION

Posterior urethral valve (PUV) is a common cause of bladder outlet obstruction in male children. Vesical calculi are commonly seen in developing countries in pediatric age group patients belonging to poor economic status. There are various congenital and acquired causes for bladder calculi. There are very few reports of association between the PUV and bladder calculi. We report three cases of vesical calculus associated with PUV.

## CASE REPORTS

### Case 1

A five-year-old boy presented with dysuria and urine dribbling for two years. He had a history of intermittent fever. Physical examination was unremarkable. There was no palpable stone on per rectal examination. X-ray KUB revealed no radio-opacity. Abdominal ultrasound was suggestive of two bladder stones 1 × 1.5 cms and 1×1 cms respectively with bilateral hydronephrosis and hydroureter. Serum creatinine was 0.7 mg %. Cystoscopy, done in view of symptoms of bladder outlet obstruction associated with dilated upper urinary tract, revealed posterior urethral valve. The child underwent fulguration of PUV, circumcision and suprapubic cystolithotomy. Postoperatively, the patient had good urinary stream. Urinary stone analysis revealed calcium, phosphate, oxalate and urate as main constituents. Follow-up micturating cystourethrogram (MCU) and cystoscopy were done which revealed normal anatomy. The patient has been on follow-up for more than two years with no complaints.

### Case 2

A four-year-old boy presented with complaints of dribbling and frequency. The patient had no similar complains in the past. Physical examination revealed phimosis. Plain X-ray abdomen didn't reveal any radioopaque density in the bladder region. Sonography was suggestive of vesical calculus with normal urinary tract. The patient underwent MCU in view of bladder obstruction which revealed dilated posterior urethra with stone. The patient was taken for cystoscopy which confirmed posterior urethral valve and vesical calculus of size 2 cm × 2 cms.

The patient underwent fulguration of PUV, circumcision and suprapubic cystolithotomy. Chemical analysis of stone revealed calcium, xanthine and oxalate. Postoperatively, follow-up MCU showed no evidence of residual posterior urethral valve. The patient is on follow-up for more than six months with no complaints.

### Case 3

A five-year-old boy was admitted with dribbling and strangury. The patient had no past history of poor urinary stream. On rectal examination, a stone was felt and the posterior urethra was dilated. The patient underwent plain x-ray abdomen which revealed bladder calculus of 5 cm × 5 cm size. Sonography confirmed calculus but could not comment on urethra. MCU revealed dilated posterior urethra. The patient underwent fulguration of PUV and suprapubic cystolithotomy. Chemical analysis of stone revealed calcium, xanthine, phosphate and oxalate. At three months follow-up, the child was asymptomatic and a repeat MCU showed no evidence of posterior urethral valve.

## DISCUSSION

PUV is a common disease. Patients usually present as neonates with bladder outlet obstruction-poor urinary stream and urinary tract infection.[1] PUV may rarely be diagnosed during later childhood, adolescence or even adulthood.[2] The common symptoms in later age group are repeated urinary tract infection or nocturnal enuresis.[3] Poor urinary stream is seen in only 10% of cases which present late. Vesical calculus is a common entity seen in children in the developing world and presents with frequency and hematuria. Vesical calculus can be associated with the congenital anomalies of urinary tract.[4] It is commonly diagnosed on plain x-ray abdomen but 10% of calculi are radiolucent which are missed on radiology. Sonography can also miss calculus in some cases. There are very few reports of association between PUV and vesical calculus.[1,5] There is a report of calculi associated with PUV in a 14-year-child.[6]

As the clinical manifestations are overlapping, PUV may be missed in case of vesical calculus as symptoms are attributed to the vesical calculus.[7] There is a report on posterior urethral valve and bladder calculus found at the age of 32 years.[2]

Sonography, in the first case, revealed vesical stone and in the second case stone was detected on micturating cystourethrogram. In case I the posterior urethral valve was detected incidentally on cystoscopy as the patient had not undergone preoperative MCU. Posterior urethral valve leads to stasis of urine and bladder dysfunction which can lead to calculus formation. Dilatation at bladder neck can occur due to impacted stone but can be differentiated on MCU from posterior urethral valve. Routinely, patients presenting with dribbling and dysuria undergo a plain abdomen X-ray, an ultrasound, and cystoscopy. As it is not feasible to do MCU in all patients with vesical calculus, we propose that children with sonography findings suggestive of upper tract dilatation or dilated posterior urethra should undergo MCU to rule out posterior urethral valves. Patients presenting with vesical calculus at age group other than common should undergo either MCU or cystoscopy to find this rare association. If symptoms persist after vesicolithotomy, MCU or cystoscopy should be done to rule out associated PUV which was missed.[7] Cystoscopy, fulguration of the valve and vesicolithotomy will correct the patient's symptoms and signs; when the facilities of endoscopic management of vesical calculus is available it should be preferred In conclusion patients of vesical calculus with symptoms of bladder outlet obstruction and dilated posterior urethra on sonography or MCU, PUV need high index of suspicion for diagnosis and further management.

## References

[1] Neulander E, Kaneti J (1996). Posterior urethral valves and vesicolithiasis in children. Int Urol Nephrol.

[2] Barroso U, Macedo A, Srougi M (2000). Posterior urethral valve in adult. Int Braz J urol.

[3] Bomalaski MD, Anema JG, Coplen DE, Koo HP, Rozanski T, Bloom DA (1999). Delayed presentation of posterior urethral valves: a not so benign condition. J Urol.

[4] Mshelbwala PM, Ameh EA, Mbibu HN (2005). Urinary stones in children. Nigerian J Surg Res.

[5] Suhler A, Masson JC, Blanchard J (1974). Valve of posterior urethra complicated by extensive vesicolithiasis. J Urol Nephrol (Paris).

[6] Mallouh C (1993). Urethral valves: Unusual presentation in 14-year-old boy. Int Urol Nephrol.

[7] Sinha A, Sarin YK, Sengar M (2001). Posterior urethral valves associated with urethral calculi. Indian J Urol.

